# ZnO–Polyaniline Nanocomposite Functionalised with Laccase Enzymes for Electrochemical Detection of Cetyltrimethylammonuium Bromide (CTAB)

**DOI:** 10.3390/jox14040106

**Published:** 2024-12-16

**Authors:** Hilda Dinah Kyomuhimbo, Usisipho Feleni, Nils Hendrik Haneklaus, Hendrik Gideon Brink

**Affiliations:** 1 Department of Chemical Engineering, University of Pretoria, Pretoria 0028, South Africa; u21830658@tuks.co.za; 2 Institute for Nanotechnology and Water Sustainability (iNanoWS), College of Science, Engineering and Technology, University of South Africa, Johannesburg 1709, South Africa; felenu@unisa.ac.za; 3 Td Lab Sustainable Mineral Resources, University for Continuing Education Krems, Dr.-Karl-Dorrek-Straße 30, 3500 Krems an der Donau, Austria; nils.haneklaus@donau-uni.ac.at; 4 Unit for Energy and Technology Systems—Nuclear Engineering, North-West University, 11 Hoffman Street, Potchefstroom 2520, South Africa

**Keywords:** cationic surfactants, metal–polymer composite, immobilisation, electrodeposition

## Abstract

The direct discharge of cationic surfactants into environmental matrices has exponentially increased due to their wide application in many products. These compounds and their degraded products disrupt microbial dynamics, hinder plant survival, and affect human health. Therefore, there is an urgent need to develop electroanalytical assessment techniques for their identification, determination, and monitoring. In our study, ZnO-PANI nanocomposites were electrodeposited on a glassy carbon electrode (GCE), followed by the immobilization of laccase enzymes and the electrodeposition of polypyrrole (PPy), to form a biosensor that was used for the detection of CTAB. A UV-Vis analysis showed bands corresponding to the π-π* transition of benzenoid and quinoid rings, π-polaron band transition and n-π*polaronic transitions associated with the extended coil chain conformation of PANI, and the presence and interaction of ZnO with PANI and type 3 copper in the laccase enzymes. The FTIR analysis exhibited peaks corresponding to N-H and C-N stretches and bends for amine, C=C stretches for conjugated alkenes, and a C-H bend for aromatic compounds. A high-resolution scanning electron microscopy (HRSEM) analysis proved that PANI and ZnO-PANI were deposited as fibres with hairy topography resulting from covalent bonding with the laccase enzymes. The modified electrode (PPy-6/GCE) was used as a platform for the detection of CTAB with three linear ranges of 0.5–100 µM, 200–500 µM, and 700–1900 µM. The sensor displayed a high sensitivity of 0.935 μA μM^−1^ cm^−2^, a detection limit of 0.0116 µM, and acceptable recoveries of 95.02% and 87.84% for tap water and wastewater, respectively.

## 1. Introduction

Cationic surfactants have numerous industrial applications due to their unique properties of emulsification, dispersion, and surface or biological activity. They can be found in many products, including household and cleaning agents, and as a result, they are often discarded into wastewater streams [1]. The toxicity of the surfactants, coupled with the short solids retention time and the impaired treatment performance of wastewater treatment plants, ultimately leads to the release of pollutants into the environment [2,3]. In addition to their toxicity, once in the environment, they persist and act as transfer systems for the aqueous solution of other pollutants such as petroleum products, pesticides, and organochlorine compounds [4,5]. As a result, antimicrobial resistance genes (ARGs) can be produced in the microorganism community [2,3].

Cetyltrimethylammonium bromide (CTAB), which is classified as one of the most toxic surfactants, is widely used in dispersants [6], coating materials [7,8], antiseptics, detergents, and fabric softeners [4]. Its main disadvantage is its poor biodegradability in the environment and high level of toxicity, which makes it toxic to aquatic systems and causes chronic health problems [9]. Its occurrence in the environment has been reported to be up to 49 µg/L in wastewater [10]. Due to its cationic nature, it easily adsorbs onto negatively charged cell surfaces, leading to cell membrane disruption and eventually cell death [11,12,13]. CTAB has been found to inhibit microorganisms from utilising readily degradable carbon sources [14], causing mitochondria dysfunction at the subcellular level [15], reducing root growth in plants [16], and inducing oxidative stress in cells [17,18], even at very low concentrations. Hence, monitoring its presence in the environment is crucial.

Classical methods for determining the presence of CTAB include high-performance liquid chromatography, two-phase titration, capillary electrophoresis, and flow injection analysis [19]. These methods are expensive and time-consuming and require skilled personnel and toxic solvents for extraction. Recent research has focused on the design and construction of biosensors that can improve the efficiency of site monitoring [20]. Enzyme-coupled bio-electrochemical systems have received attention due to their easy handling, quick response processes, basic instrumentation, and selective monitoring under mild conditions [21,22]. Laccase has been explored in biosensor applications due to its ability to oxidise a variety of organic substrates [23,24]. The ability to catalyse electron transfer reactions without additional cofactors makes laccase a superior bioreceptor in sensor applications compared to other enzymes [25,26].

Different materials and techniques have been explored for the immobilisation of enzymes in sensor applications [27,28]. Conductive polymer materials used as holding matrices for immobilised enzymes have been explored in biosensor construction due to their low cost, flexibility, and biocompatibility [29,30].

Polyaniline (PANI) has gained attention in enzymatic biosensor construction due to its low cost, its ease of synthesis from an aniline monomer, and the ease of changing its electronic structure through de-doping using its high electrical conductivity, high surface area, and ease of deposition on electrodes [31,32]. Since immobilisation might reduce the enzymatic activity, other materials can be coupled to counteract this effect. Nanoparticles (NPs) can be incorporated into an enzyme–polymer matrix to increase the sensitivity and overall performance of the biosensor [33,34]. NPs improve the electron transfer efficiency between the redox active sites of the enzyme and electrode; hence, they are ideal for combination with polymers that minimise the disadvantages and maximise the advantages of each individual support material [35]. ZnO nanoparticles (ZnONPs) have been exploited as a potential material for enzyme immobilisation and biosensing due to their beneficial features, such as their high surface area for strong adsorption and effective enzyme loading, chemical stability, good biocompatibility, high electron communication, and lack of toxicity [36]. The divalent nature of Zn^2+^ can also improve laccase activity through competition with Cu^2+^ in the electron transport system, which improves the laccase–substrate relationship [28,37].

In this study, laccase enzymes were immobilised on an electrodeposited ZnO-PANI GCE modified electrode. A thin film of polypyrrole (PPy) was electrodeposited on top of the laccase enzyme to prevent enzyme leakage. The PPy-Lac-ZnO-PANI/GCE was then used to detect CTAB in PBS, tap water, and wastewater. To the best of our knowledge, no studies on the use of enzymatic biosensors for the detection of CTAB have been reported in the literature. Unlike the traditional techniques used to measure levels of CTAB, the biosensor designed and reported in this study is easy to use, does not require skilled personnel, can provide continuous real-time quantification of CTAB with high sensitivity, and can detect very low concentrations that are environmentally relevant. The biosensor demonstrated good performance in tap water and wastewater and can therefore find applications in environmental monitoring.

## 2. Materials and Methods

Zinc sulphate (ZnSO_4_·7H_2_O, 99%), aniline (C_6_H_5_NH_2_, 99.5%), hydrochloric acid (HCl, 37%), pyrrole (C_4_H_5_N, 98%), cetyltrimethylammonium bromide (CTAB) (CH_3_(CH_2_)_15_N(Br)(CH_3_)_3_, 98%), glutaraldehyde (C_5_H_8_O_2_, 50%), and laccase enzymes (from *Aspergillus* sp.) were obtained from sigma Aldrich, Johannesburg, South Africa.

### 2.1. Electrodeposition of ZnO-PANI Nanocomposite

A single-compartment cell equipped with a three-electrode electrochemical system was utilised for all electrodeposition studies. The system consisted of a platinum (Pt) wire as the counter electrode, a glassy carbon electrode (GCE) as the working electrode, and a Ag/AgCl (3 M NaCl) electrode as the reference. Before the electrodeposition of the composite, the GCE was meticulously polished using alumina slurries of 1.0, 0.3, and 0.05 μm, followed by sonication in methanol and deionised water to ensure thorough cleaning. The working solution for the electro-polymerisation of PANI film contained 0.1 M aniline and 1 M hydrochloric acid (HCl). To incorporate ZnO in PANI, the aniline solution was mixed with varying concentrations of ZnSO_4_ and stirred at 400 rpm to ensure complete dissolution. Different concentrations of ZnSO_4_ were added to investigate the effect of metal loading on the electrical conductivity of the composite. The solution was degassed by bubbling nitrogen gas through it for 7 min prior to the start of the electro-polymerisation process. A nitrogen gas blanket was subsequently maintained above the solution throughout the electrodeposition and characterisation processes. PANI and ZnO-PANI films were electrodeposited onto a GCE from a 1 M HCl solution containing 0.2 M aniline and 0.1 M ZnSO_4_ using voltammetry. The potential was swept between −0.2 V and 1.2 V versus SCE at a scan rate of 50 mV/s for 40 cycles. The PANI- and ZnO-PANI-modified electrodes (PANI|GCE and ZnO-PANI|GCE) were thoroughly rinsed with distilled water to remove any excess monomer and characterised in 1 M HCl using CV from −1 V to 1.2 V at varying scan rates.

### 2.2. Modification of the Electrodes with Laccase Enzyme and Detection of CTAB

To immobilise the enzyme, 5 µL of laccase was applied to the ZnO-PANI-modified electrode, followed by the addition of 5 µL of 2% glutaraldehyde in 0.1 M phosphate buffer solution (PBS). The glutaraldehyde acted as a crosslinking agent between the composite and laccase enzyme. The electrode was left to stand overnight at 4 °C and subsequently rinsed with 0.1 M PBS. To prevent the enzyme leakage, a protective polypyrrole layer was applied by electrodepositing 0.15 M pyrrole solution in 0.1 M PBS using CV over a potential range of −0.7 to 0.8 V at a scan rate of 50 mV/s for 15 cycles. The solution was oxygenated by bubbling oxygen for 3 min prior to CV. The activity of the electrode was evaluated using CV at different pH levels to determine the optimum working pH in 0.1 M PBS. Differential pulse voltammetry (DPV) was conducted on the PPy-Lac-ZnO-PANI/GCE-modified electrode in the presence of CTAB at varying concentrations in 0.1 M PBS.

### 2.3. Characterization of Electrode and Nanocomposite

The surface plasmon resonance (SPR) properties and spectral characteristics of PANI, ZnO-PANI, and PPy-Lac-ZnO-PANI were examined using a UV–visible spectrometer (UV-1600PC; Avantor, Apeldoorn, The Netherlands) across the wavelength range of 250 to 900 nm. Additionally, Fourier Transform Infrared (FTIR) spectroscopy (Bruker Alpha II Platinum ATR, Bruker, Coventry, UK) was performed over the range of 4000 to 400 cm^−1^ with 25 scans. FTIR was used to identify the functional groups present in the nanocomposite. High-resolution scanning electron microscopy (HRSEM) analysis was performed on electrodes that were electrodeposited and modified on a screen-printed carbon electrode (SPCE). The bare and modified SPCE electrodes were characterised using HRSEM to analyse their morphological properties. Electrochemical characterisation was conducted using a Palm Sens Emstat3+ Blue potentiostat (Palm Sens BV, Houten, the Netherlands). The electrochemical behaviour of PANI, ZnO-PANI, and PPy-Lac-ZnO-PANI was investigated using CV at a scan rate of 10 mV/s. Experiments on PANI and ZnO-PANI were conducted in 1 M HCl within a potential window of −1.0 to 1.2 V starting with an anodic sweep from −1.0 V. In contrast, the PPy-Lac-ZnO-PANI electrode was analysed in 0.1 M PBS within a potential range of −0.7 to 1.2 V. The performance of the laccase-based sensor was evaluated in 0.1 M PBS at varying pH levels to identify the optimum operating pH.

## 3. Results and Discussion

### 3.1. Characterisation of PPy-Lac-ZnO-PANI/GCE Nanocomposite

#### 3.1.1. UV-Vis Analysis

**Figure 1:**
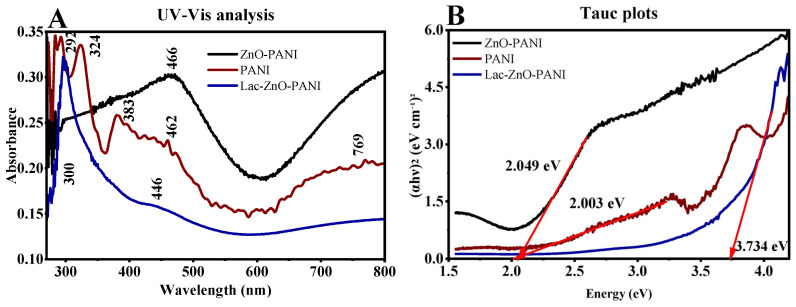
A graph of (**A**) UV-Vis analysis and (**B**) Tauc plots for PANI and ZnO-PANI and PPy-Lac-ZnO-PANI composites.

Surface plasmon resonance (SPR) is a distinctive optical phenomenon exhibited by metallic nanoparticles, where the surface plasmon oscillations of free electrons on the NP surface are induced by incident photons. This interaction leads to the absorption of light by the NPs, which can be detected by UV-Vis spectroscopy. The characteristics of the SPR band are influenced by several factors, including the metal type, the NPs’ size and shape, and the surrounding medium [38]. To analyse the composite using UV-Vis spectroscopy (Figure 1A), the electrode-deposited materials—PANI, ZnO-PANI, and PPy-Lac-ZnO-PANI—were redispersed in 2 mL of 2 M HCl and transferred to a cuvette. PANI exhibited three distinct SPR bands at 292, 324, and 383 nm, along with two broad bands at 462 and 769 nm. The band at 292 nm is likely attributed to the π-π* transition of benzenoid and quinoid rings [39], while the bands at 324 nm and 383 nm may correspond to π-π* electron transitions associated with the fundamental structural units of PANI chains [40]. The band at 462 nm may be attributed to the π-polaron band transition at this wavelength or to the oxidised dimer of aniline, diphenyl-p-phenylenediamine, which absorbs at 464 nm [41]. The broad free-carrier band at 769 nm, extending towards the near-infrared region, is ascribed to n-π* polaronic transitions. These transitions are associated with the transformation of PANI from a compact coil to an extended coil chain conformation as the emeraldine salt form develops [42,43]. The addition of zinc sulphate to the monomer to form a ZnO-PANI nanocomposite resulted in the disappearance of all the bands in the 200–400 nm range. However, a pronounced increase in the excitonic transition peak around 466 nm was observed, confirming the presence of and interaction between ZnO and the PANI molecule [44]. A blue shift in the broad band towards the near-infrared region was also noted, likely due to electrostatic interactions between ZnO and PANI chains, leading to electron transfer from the LUMO band of PANI molecules to the conduction band of the ZnO lattice [45]. With the introduction of the laccase enzyme, a new band emerged at 300 nm, corresponding to the type 3 Cu centre of the laccase enzyme [46,47].

Tauc plots (Figure 1B) were employed to determine the energy band gap of PANI, ZnO-PANI nanocomposites, and PPy-Lac-ZnO-PANI nanocomposites, which were found to be 2.003 eV, 2.049 eV, and 3.734 eV, respectively. The addition of ZnO nanoparticles to PANI clearly altered the energy band gap of PANI, likely due to charge transfer transitions. This change can be attributed to the mobile electrons in the ZnO nanoparticles causing electrostatic interactions, as well as the creation of new excitation energy levels resulting from electron transfer from the ZnO nanoparticles to the PANI matrix [48,49].

#### 3.1.2. FTIR

**Figure 2:**
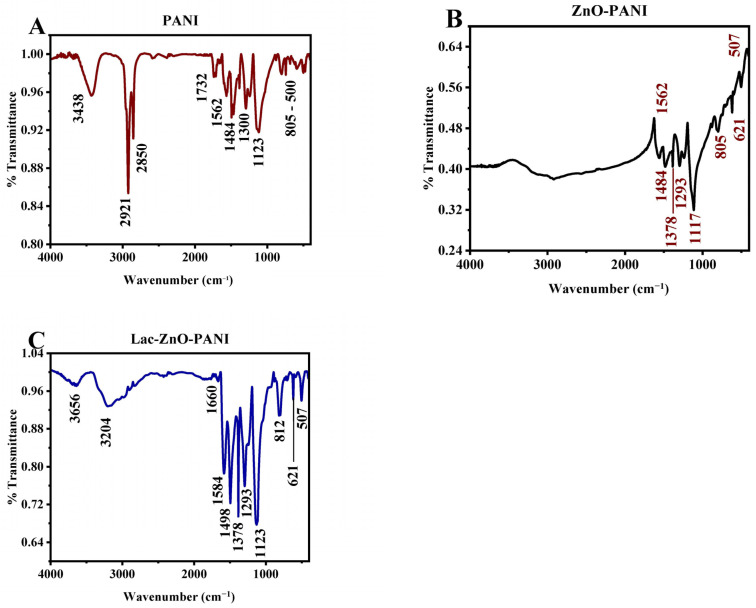
FTIR spectra for (**A**) PANI and (**B**) ZnO-PANI and (**C**) PPy-Lac-ZnO-PANI composites.

FTIR spectra for PANI, ZnO-PANI, and PPy-Lac-ZnO-PANI were recorded in the range of 4000 to 400 cm^−1^ (Figure 2A–C) to identify the functional groups present. PANI exhibited characteristic peaks at 3438 cm^−1^ and 2921 cm^−1^, corresponding to N-H stretching of amines in the polymer structure; 2850 cm^−1^ for C-H stretching of alkenes; 1732 cm^−1^ for the C-H bending overtone of aromatic compounds; 1562 cm^−1^ for C=C stretching of conjugated alkenes in quinoid and benzenoid rings; 1484 cm^−1^ for N-H bending of amines; 1300 cm^−1^ and 1123 cm^−1^ for C-N stretching associated with N=Q=N in the quinoid ring; and peaks at 805 cm^−1^, 741 cm^−1^, 684 cm^−1^, 592 cm^−1^, and 507 cm^−1^ for C=C bending of aromatic rings [50,51,52].

Similarly, ZnO-PANI exhibited characteristic peaks at 1562 cm^−1^ for C=C stretching of conjugated alkenes; 805 cm^−1^, 621 cm^−1^, and 507 cm^−1^ for C=C bending of alkenes; 1484 cm^−1^ for N-H bending of amines; and 1378 cm^−1^, 1293 cm^−1^, and 1117 cm^−1^ for C-N stretching of amines [53,54,55]. In the case of PPy-Lac-ZnO-PANI, peaks were observed at 3656 cm^−1^, 3204 cm^−1^, and 1498 cm^−1^ for stretching of primary and secondary amines; 1584 cm^−1^, 812 cm^−1^, 621 cm^−1^, and 507 cm^−1^ for C=C stretching and bending of alkenes; and 1293 cm^−1^, and 1123 cm^−1^ for C-N stretching of aromatic amines [56]. The absence of the peak at 1715 cm^−1^, corresponding to the aldehyde group, indicates complete crosslinking between the enzyme and polymer composite [57]. Furthermore, according to Thiyagarajan et al. [58], free laccase exhibits a distinctive peak at 1664 cm^−1^, corresponding to a CO-NH bond. The absence of this peak in the modified electrode further confirms the successful crosslinking of the enzyme with glutaraldehyde.

#### 3.1.3. Scanning Electron Microscopy

**Figure 3:**
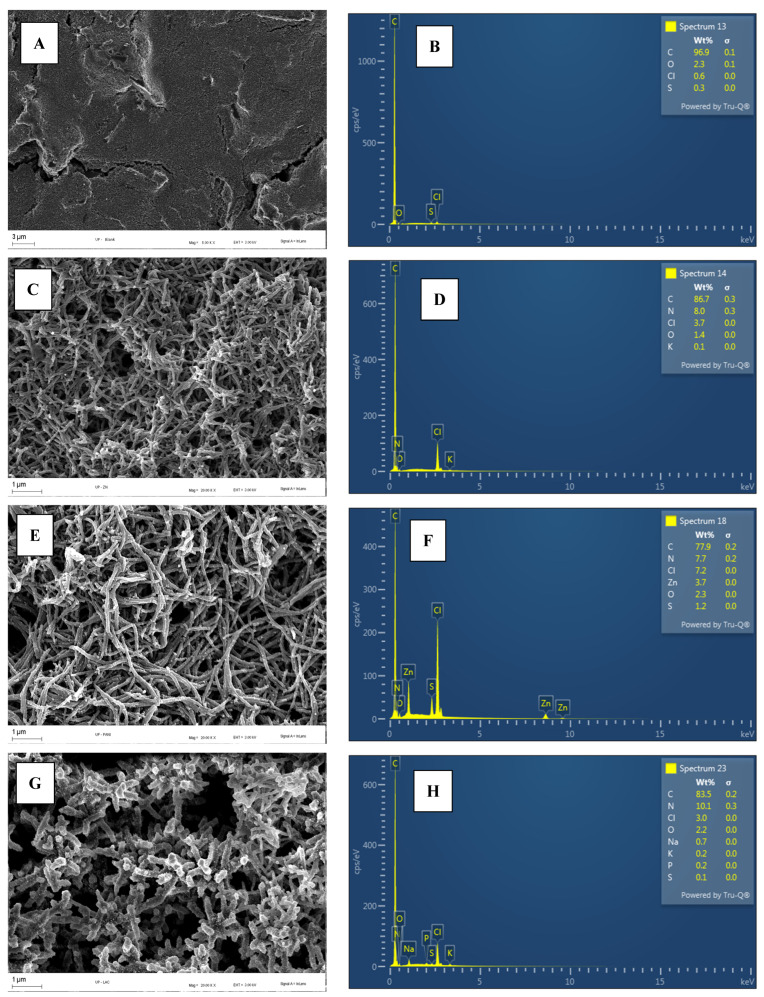
SEM and EDS for (**A**,**B**) blank SPCE and (**C**,**D**) PANI-, (**E**,**F**) ZnO-PANI-, and (**G**,**H**) PPy-Lac-ZnO-PANI-modified SPCEs.

SEM analysis was performed on screen-printed carbon electrodes (SPCEs), with Figure 3A showing the surface of a typical SPCE. The surface topography revealed that the growth of PANI from the aniline medium resulted in the formation of PANI fibres on the SPCE (Figure 3B). The incorporation of ZnO into PANI reduced the diameter of the fibres, thereby providing a larger surface area for enzyme immobilisation (Figure 3C). Enzyme immobilisation onto the nanocomposite fibres was evident from the increased fibre diameter and a change in the surface topography from smooth to hairy (Figure 3D). The hairy topography likely represents clusters of proteins on the surface of the ZnO-PANI fibres [59].

The composition of the different electrodes was analysed using energy-dispersive X-ray spectroscopy (EDS) (Figure 3E–H). Elemental C and N were detected in PANI (Figure 3F), while elemental Zn, O, C, and N were identified in ZnO-PANI (Figure 3G), confirming the formation of the ZnO-PANI nanocomposite. Following the addition of the laccase enzyme, additional elements such as P, Na, and K appeared in the sample (Figure 3H), likely attributed to the secondary and tertiary structures of the laccase protein [60]. S was also present, potentially as an impurity from the formation of zinc sulphate hydroxide trihydrate intermediate during ZnO nanoparticle formation [61].

#### 3.1.4. Electrochemical Characterisation of Nanocomposites

**Figure 4:**
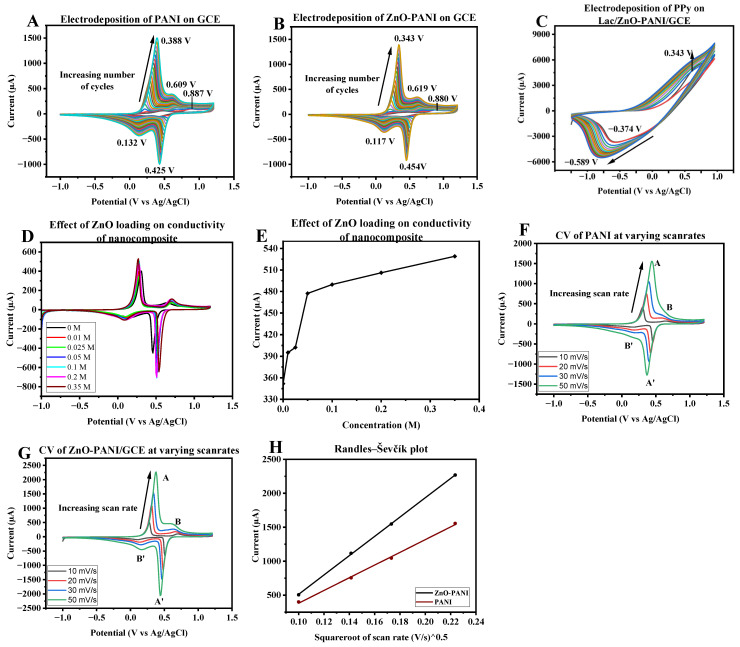
Graphs showing electrochemical deposition of (**A**) PANI, (**B**) ZnO-PANI, and (**C**) PPy; (**D**,**E**) effect of ZnO loading on the conductivity of PANI; CV of (**F**) PANI and (**G**) ZnO-PANI at varying scan rates; and (**H**) Randles–Ševčík plot for PANI and ZnO-PANI composites.

A cyclic voltammetric sweep in the potential range of −1.2 to 1.2 V for 40 cycles resulted in the oxidation of aniline and Zn^2+^ with aniline, forming dark green films of PANI and ZnO-PANI, respectively, on the electrode surfaces (Figure 4A,B). The increase in current peaks with the number of scans indicated the formation of uniform polymer/composite films on the electrode surfaces through the polymerisation process. Similarly, during the electrodeposition of polypyrrole (PPy) (Figure 4C), the current density increased with the number of scan cycles, attributed to the nucleation of PPy on the electrode [62].

The CVs for PANI and ZnO-PANI (Figure 4A,B) exhibited three distinctive redox peaks: O^1^R^1^ (0.388 V, 0.425 V), O^2^R^2^ (0.609 V, 0.132 V), and a smaller O^3^ (0.88 V). O^1^R^1^ corresponds to the conversion of leucoemeraldine to the partly oxidised emeraldine form [63]. O^2^R^2^ is attributed to secondary products such as benzoquinones and hydroquinones [64]. O^3^ represents the conversion of emeraldine to the fully oxidised pernigraniline form of PANI [65]. A shift towards a more positive potential was observed with an increasing number of cycles, attributed to the expansion of the PANI surface and an increase in current density [66].

The effect of Zn^2+^ concentration in the monomer solution on the conductivity of the nanocomposites was investigated. The results demonstrated an exponential increase in the conductivity of the nanocomposite at lower Zn^2+^ concentrations until it plateaued (Figure 4D,E). Beyond this point, further increases in Zn^2+^ concentration did not result in a proportional rise in the peak current of the nanocomposite. Consequently, 0.1 M ZnSO_4_ was selected as the optimal concentration for subsequent analyses. To validate the enhancement in conductivity on the nanocomposite by ZnO, the diffusion coefficients of H^+^ across the two modified electrodes were determined using the Randles–Ševčík Equation (1).

(1) $$i_{p}=0.4463\;nFA\;{\left[\frac{nF}{RT}\right]}^{\frac{1}{2}}\;C{D_{H^{+}}}^{\frac{1}{2}}V^{\frac{1}{2}}$$

where *i_p_* is the peak current in *A*, *n* = 1 is the number of electrons transferred, *F* = 96,485 C mol^−1^ is Faraday’s constant, *A* = 0.071 cm^2^ is the surface area of the electrode, *R* = 8.314 J mol^−1^ K^−1^ is the gas constant, *T* = 289.15 K is the temperature, ν is the scan rate in V/s, *C* is the concentration of the electrolyte in mol cm^−3^, and *D_H_*^+^ is the diffusion coefficient of the H^+^ in cm^2^ s^−1^. Using the anodic peaks (*A*) from Figure 4F,G, a plot of peak current vs. square root of the scan rate was constructed for PANI and ZnO-PANI at varying scan rates. The slope of this plot was used to calculate the diffusion coefficient (*D_H_*^+^) of H^+^ across the modified electrodes (Figure 4H). The diffusion coefficients were determined to be 1.9 × 10^−3^ cm s^−1^ for PANI and 2.9 × 10^−3^ cm s^−1^ for ZnO-PANI. The increase in *D_H_*^+^ indicates that ZnONPs enhance the electrochemical performance of PANI, as evidenced by the increased current signals (Figure 4F,G) [67].

**Figure 5:**
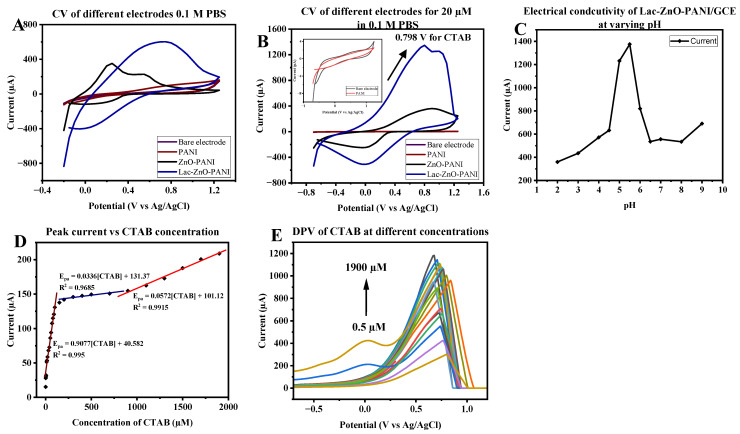
Cyclic voltammograms of different electrodes in the (**A**) absence and (**B**) presence of 20 µM CTAB in 0.1 M PBS. (**C**) A graph of electrode activity (peak current) at varying pH of the solution. (**D**) Effect of pH on conductivity of electrode and peak current vs. concentration of CTAB in 0.1 M PBS. (**E**) DPV of CTAB at different concentrations in PBS.

For further analysis, CV was employed to evaluate the electrocatalytic properties of PANI, ZnO-PANI, and PPy-Lac-ZnO-PANI in a 0.1 M PBS (pH = 7.4) electrolyte solution. Figure 5A and Figure 5B present the CVs recorded in the absence and presence of CTAB, respectively. Since the total current in CV consists of both non-faradaic (background) current and faradaic current, Figure 5A illustrates the non-faradaic current generated by the different modified electrodes in the absence of the analyte (20 µM CTAB). The background current increases in the following order: bare electrode < PANI-modified electrode < ZnO-PANI-modified electrode < PPy-Lac-ZnO-PANI-modified electrode. This increase is likely attributed to enhanced capacitance and double-layer charging effects caused by the accumulation of positively and negatively charged ions on the electrode surface [68].

In the presence of CTAB, a characteristic peak appears around 0.75 V, consistent with findings in the literature (Figure 5E). During the electrochemical process, CTAB molecules adsorb onto the electrode surface, forming a stable layer of R(CH_3_)_3_N^+^, which undergoes reduction, resulting in the cathodic peak. The anodic peak corresponds to the oxidation of Br^−^ ions [69].

The redox behaviour of the PPy-Lac-ZnO-PANI-modified electrode in 0.1 M PBS solution was examined at different pH values. The results showed that the electrode activity increased with rising pH up to 5.5 but then drastically decreased at higher pH levels (Figure 5C). To achieve a higher response signal, the optimum pH (5.5) was used for preliminary studies to detect CTAB in PBS.

**Scheme 1:**
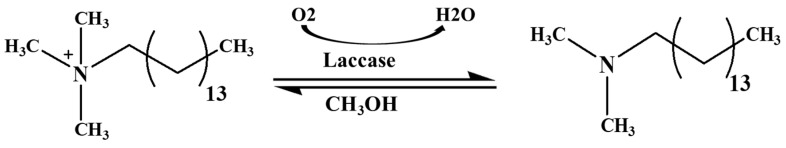
Suggested mechanism for electrochemical oxidation of CTAB [70,71].

The mechanism of interaction between the laccase enzyme and CTAB remains a subject for further investigation. However, it has been suggested that oxygen is necessary for the enzymatic degradation of CTAB [70]. Additionally, it has been proposed that interaction occurs between the cationic head group of CTAB and the negatively charged laccase enzyme, leading to demethylation [70,71] (Scheme 1).

The electrocatalytic capability of PPy-Lac-ZnO-PANI/GCE was evaluated using differential pulse voltammetry (DPV) with varying concentrations of CTAB, ranging from 0.5 µM to 1900 µM, in 0.1 M PBS (pH = 5.5) at a scan rate of 10 mV/s (Figure 5D). The results indicate that the peak current of PPy-Lac-ZnO-PANI increases linearly with CTAB concentration between 0.5 µM and 100, attributed to fast electron transfer kinetics. This demonstrates the excellent electrocatalytic performance of the PPy-Lac-ZnO-PANI/GCE for the electrooxidation of CTAB, with linear detection ranges of 0.5–100 µM, 200–500 µM, and 700–1900 µM. Three regression equations (Equations (2)–(4)) with correlation coefficients were obtained:

(2) $$i_{pa}=0.9077\left[CTAB\right]+40.582$$

(3) $$i_{pa}=0.0336\left[CTAB\right]+131.37$$

(4) $$i_{pa}=0.0572\left[CTAB\right]+101.12$$

where [*CTAB*] is the concentration of CTAB in μM and *i_p_* is the peak current in μA.

**Figure 6:**
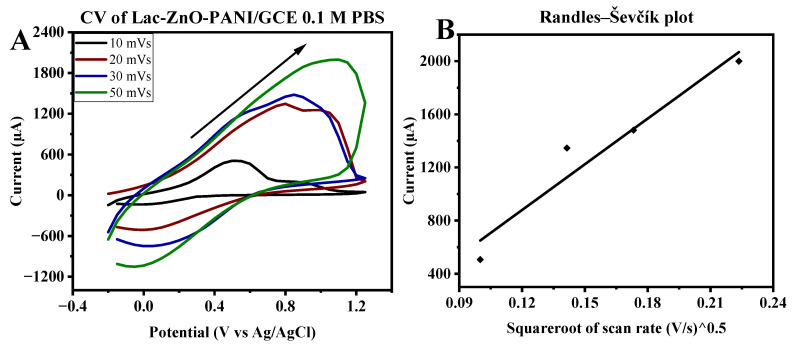
(**A**) Cyclic voltammogram of PPy-Lac-ZnO-PANI/GCE electrode in 0.01 M ABTS in 0.1 M PBS and (**B**) Randles–Ševčík plot for PPy-Lac-ZnO-PANI/GCE in 0.01 M ABTS in 0.1 M PBS.

To determine the sensitivity, the equation for the lower concentration range was used. CV was performed on the PPy-Lac-ZnO-PANI/GCE in the presence of 0.01 M ABTS in 0.1 PBS (pH = 5.5) at varying scan rates (Figure 6A). The Randles–Ševčík Equation (1) was applied to estimate the active surface area of the electrode, using the slope obtained from Figure 6B. Assuming the diffusion coefficient of ABTS in 0.1 M PBS is 4.4 × 10^−4^ cm^2^ s^−1^ [72], the active surface area of PPy-Lac-ZnO-PANI was calculated as 0.9703 cm^2^_._ The sensitivity of the electrode was determined as 0.935 μA μM^−1^ cm^−2^. Using Equation (5), the limit of detection was calculated as 0.0116 μM.

(5) $$LOD=\frac{3\sigma }{q}$$

where *σ* represents the standard deviation of the background and *q* is the slope obtained from the calibration curve at lower concentrations.

#### 3.1.5. Biosensor Performance in Presence of Interferents and Real Samples and Reusability Studies

To evaluate the practical applicability of a biosensor, selectivity is a critical parameter. Selectivity is one of the key advantages of biosensors, allowing the detection of specific analytes within complex mixtures without the need for prior separation [73]. In this study, the selectivity of the biosensor was assessed by identifying potential interferents that could undergo oxidation or reduction with laccase at similar potentials. These compounds were chosen based on their potential to influence the biosensor’s signal.

The biosensor was tested for its ability to detect the target analyte, CTAB, in the presence of these interferents, which were introduced at significantly higher concentrations relative to CTAB. The ability of the biosensor to recognise CTAB in the presence of other competing compounds is essential for its practical application [74]. Two possible interferents, benzalkonium chloride (BAC) (100 µM) and hexamethylenetetramine (HEX) (100 µM), were used in this study alongside CTAB (20 µM).

**Figure 7:**
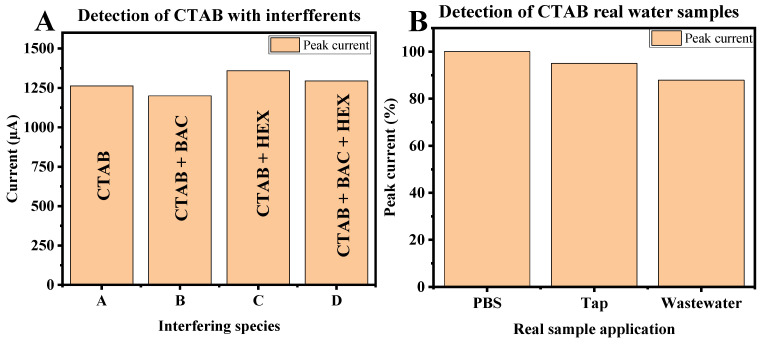

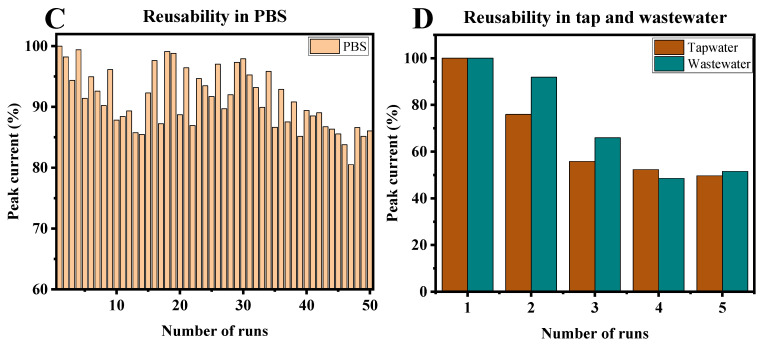
Performance of the PPy/Lac-ZnO-PANI/GCE in presence of (**A**) interferants and (**B**) in real water samples and reusability of the biosensor in (**C**) PBS and (**D**) in tap water and wastewater.

According to Figure 7A, the voltammetric oxidation signal of CTAB was not significantly affected by the presence of these interferents, although a slight shift in the peak potential was observed. This shift is likely due to interactions occurring at the electrode surface [68]. The relative standard deviation (RSD) was calculated as 5.24%, indicating good sensitivity of the PPy-Lac-ZnO-PANI/GCE sensor. The sensor was further tested on tap water and sewage wastewater spiked with CTAB, and no significant changes in the peak current were observed, confirming its applicability for real samples.

The wastewater was obtained from the Daspoort wastewater treatment facility in Pretoria, South Africa, and progressively filtered using membrane filters of 110, 0.45, and 0.22 µm pore sizes. The tap water and wastewater samples were diluted with 0.1 M PBS to a final concentration of 50% *v*/*v* and spiked with CTAB to achieve a concentration of 20 µM. These samples were then analysed using DPV. The recoveries were recorded as 95.02% for tap water and 87.84% for wastewater (Figure 7B).

The stability and reusability of the sensor were tested for 50 cycles spanning one week, with the electrode stored at 4 °C between tests. The voltammetric response of the sensor was compared to the initial current of the modified electrode in 20 µM CTAB. The study was conducted in triplicate using three different electrodes modified using the same procedure and evaluated using DPV. After 50 cycles over 7 days, the peak current decreased to 85.17% of its original value in 0.1 M PBS (Figure 7C), with an RSD of 5.51% across the three electrodes.

In the case of tap water and wastewater, the electrode performance declined more sharply. For tap water, the peak current reduced to 76.2% and 49.8% after the second and fifth tests, respectively, within 24 h. For wastewater, the performance dropped to 91.9% and 51.5% after the second and fifth tests, respectively, within the same time frame (Figure 7D). This significant decline is likely due to the blockage of active sites on the electrode surface by other organic components, which hinders the charge transfer process [69]. Further studies and electrode modifications are necessary to enhance the reusability of the sensor, particularly for applications in wastewater samples.

## 4. Conclusions

This study successfully developed a PPy-Lac-ZnO-PANI/GCE electrode biosensor for the detection of CTAB in real water samples, showcasing its potential for continuous, real-time monitoring applications. The biosensor, constructed by immobilising laccase enzymes onto a ZnO-PANI composite matrix, demonstrated excellent sensitivity of 0.935 μA μM^−1^ cm^−2^, high selectivity in the presence of BAC and HEX interferents, and reliable performance across a wide concentration range. The incorporation of ZnO nanoparticles significantly enhanced the sensor’s electrochemical properties by facilitating efficient electron transfer, thereby improving sensitivity.

Furthermore, the biosensor displayed remarkable stability in PBS and achieved good recovery rates for CTAB detection in both tap water and wastewater samples. Its low detection limit of 0.0116 μM highlights its capability for monitoring CTAB at environmentally relevant concentrations. This biosensor represents a promising alternative to traditional, more complex methods for CTAB detection, offering a more accessible and efficient solution for environmental monitoring.

Future studies will focus on optimising the sensor’s response and enhancing its stability in real water samples, including tap water and wastewater, to ensure its suitability for such applications.

## Data Availability

The raw data supporting the conclusions of this article will be made available by the authors upon request.
